# Calculation of Volume Fractions of In Situ TiB and Residual Stress Distributions in Functionally Graded Composite of Ti–TiB–TiB_2_

**DOI:** 10.3390/ma12234006

**Published:** 2019-12-03

**Authors:** Youfeng Zhang, Shasha He, Wanwan Yang, Jiangwei Ren, Haijuan Kong

**Affiliations:** School of Materials Engineering, Shanghai University of Engineering Science, Shanghai 201620, China; He13100619279@163.com (S.H.); yangwan1994@sina.cn (W.Y.); jwren@163.com (J.R.); khj3155@126.com (H.K.)

**Keywords:** functionally graded material (FGM), TiB, spark plasma sintering (SPS), x-ray diffraction (XRD), residual stress

## Abstract

Ti matrix composite with a polylaminate structure was successfully fabricated via spark plasma sintering (SPS) process. A temperature gradient field (TGF) was obtained during the sintering to form functionally graded material (FGM) in a vacuum under 40 MPa for 5 min. The actual volume fractions of TiB in the matrix were calculated based on the X-ray diffraction pattern. The target volume fractions of TiB were 0%, 20%, 40%, 60%, 80% and 100%. The calculated TiB volume fractions were slightly higher than the target volume fractions in layers 2–4 and lower than the target volume fractions in layers 5–6 and the deviations in layers 4 and 5 were less than 5% of the target volume. Based on the elastic axial symmetry model, the residual stress distributions in the Ti matrix composite with a polylaminate structure were simulated, indicating a relatively low thermal residual stress in the FGM.

## 1. Introduction 

The phases and components in functionally graded materials (FGMs) can be tuned to achieve small changes in their constituents, microstructure and properties in certain directions. FGMs have received wide attention in research because of their excellent performance, precise designs and extensive applications [1,2,3,4,5]. However, the vital issue in fabricating FGMs is residual stress in the samples, which results in crack or cavity formation. The residual stress is produced by mismatching of Ti alloy and other phases with different coefficient of thermal expansion (CTEs) in continuous structures. Titanium boride, especially TiB and TiB_2_, has many excellent properties, such as high hardness, melting point, conductivity and shock performance, and it is chemically inactive [6,7]. Furthermore, because of the difference in mechanical properties and similar CTEs of titanium boride (TiB) and titanium (Ti), TiB and TiB_2_ can be prepared by an in situ reaction between Ti and B or TiB_2_ [8,9]. As a type of synthesis, consolidation and processing technique spark plasma sintering (SPS) has rapidly developed in recent years. Rapid sintering at low temperatures can be achieved by charging at intervals between electrically charged sintered powders and spark plasma, which is transiently generated, at high temperatures. SPS, a type of rapid consolidation technique, has contributed to many applications in manufacturing advanced materials and FGMs [10,11,12,13]. Hence, theoretical studies on the properties and microstructures of FGMs have been extensively conducted by researchers. Zhang et al. [8] fabricated FGMs of TiB–Ti via SPS with die modification to achieve a stable graded temperature field. Feng et al. [14] obtained a Ti–TiB–TiB_2_ composite with different layer structures via SPS in vacuum. In our previous work [15], we prepared a six-layer functionally graded Ti–TiB–TiB_2_ material and investigated the microstructure and mechanical properties. A method for synthesizing Ti–TiB composite FGMs via a temperature gradient field (TGF) process was probed, which was realized via SPS, and the hardness and microstructure of each layer were studied. Based on the above research, the microstructure, mechanical properties and densification of the in situ SPS synthesized functionally graded TiB–TiB_2_ material was studied. Nonetheless, it is noteworthy that the amount of TiB generated significantly influenced the properties of the TiB composite, because many of the mechanical properties of titanium composites, such as strength, hardness and elastic modulus, improve gradually with the formation of TiB phase. Meanwhile, the stresses distribution in different layers also has a significant effect on the microstructure and properties of the materials. Numerical simulation is a common efficient method to study residual stress distribution. The mechanical properties of TiB–Ti FGMs can be indirectly predicted based on the TiB content, but the cited literature confirmed that no specific research has systematically explained the differences between the target and actual amounts of TiB phase in synthesized Ti–TiB FGMs. Herein, a Ti–TiB–TiB_2_ FGM was fabricated using Ti, TiB_2_ and B powders via an SPS process, the estimation of the TiB volume fraction was investigated, the sintering TGF and thermal residual stress in individual layers of FGM was calculated by finite element simulation.

## 2. Methods and materials

**Figure 1:**
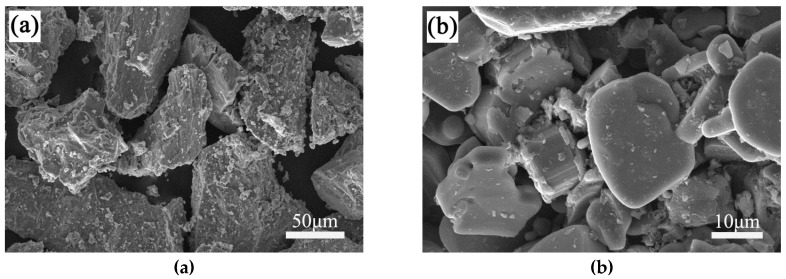
SEM micrographs of original powder particles of Ti (**a**) and TiB_2_ (**b**).

The mixtures used for the sintered FGMs comprised Ti (>99%), FeMo (>99%), B (>95%) and TiB_2_ (>99%) powder and their average sizes were 74, 74, 2, and 30 μm, respectively. The powder morphologies of Ti, TiB_2_ and B are large granular, sheet and amorphous, the scanning electron microscope (SEM) micrographs of Ti and TiB_2_ are shown in Figure 1. During SPS, Equations (1) and (2) represent the generation of TiB from Ti and B or Ti and TiB_2_, respectively. 

(1) Ti+B=TiB

(2) $$\mathrm{Ti}+{\mathrm{TiB}}_{2}=2\mathrm{TiB}$$

**Figure 2:**
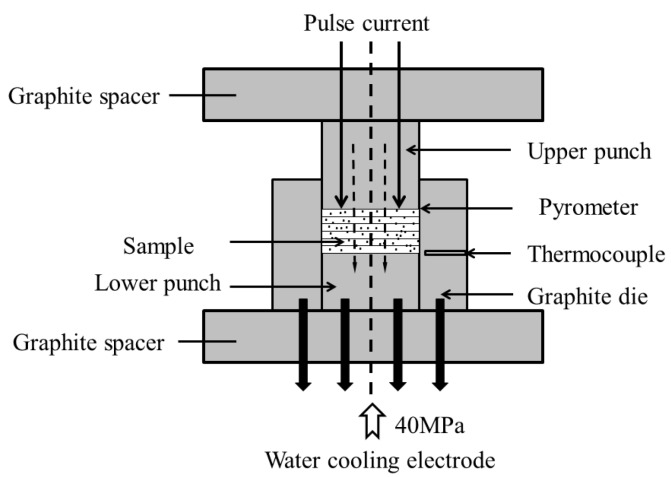
Schematic diagram of temperature gradient field (TGF) sintering by spark plasma sintering (SPS).

Different fractions of B and TiB_2_ were present in the 40, 60, 80 and 100 vol% target TiB layers, while TiB_2_ powder was applied to the 20 vol% TiB layer. The Ti matrix (Ti–4.0Fe–7.3Mo) comprised a mixture of Ti and FeMo powder. The target compositions were 0%, 20%, 40%, 60%, 80% and 100% TiB, respectively. Each powder mixture with a given target volume fraction was mixed for 10 h in a planetary ball mill. The FGM was then placed layer-by-layer from 0% to 100% TiB in a graphite die, and the radius of a fabricated sample was 10 mm for the subsequent sintering step. The temperature of upper punch was measured by means of an optical pyrometer focused on its surface. Then the compact was sintered at 950 °C with a heating rate of 150 °C /min and dwelling 5min with a pressure of 40 MPa in vacuum. Figure 2 shows a schematic of the SPS process with the generated TGF. Model of the SPS furnace was SPS-2050. At the start of the heating process, the temperature difference between the top and the bottom of the die was approximately 650 °C because of the accompanying gradient field, which was kept constant. When the temperature of the Ti-rich side reached 950 °C, the maximum temperature of the TiB-rich side accordingly reached 1569 °C. The pressure was applied from the upper punch as shown in Figure 2. There are two temperature measured points. The first point is in the middle of the graphite mould near the fist compacted layer, and the sintering temperature was controlled by this feedback information. The second point is the upper punch near the top of the mould, which is similar to the upper compact layer when sintering. The sintering temperature can be controlled and the TGF can be estimated. The TGF sintering process is explained in detail in our previous paper [15]. The X-ray direct comparison method was used the calculation of volume fractions of TiB and the integrated intensities of the X-ray peaks of a particular phase are computed based on assuming the shape of the peaks to be Gaussian. The sample microstructures were observed with a scanning electron microscope (Hitachi S-3500N, Tokyo, Japan). The ANSYS 10 software (ANSYS Inc., Canonsburg, PA, USA)was used for finite element model (FEM) modeling of the thermal residual stress distribution.

## 3. Results and Discussion

### 3.1. Calculation of Actual TiB Fraction

The actual fractions of TiB in the matrix were calculated based on the X-ray diffraction (XRD) pattern by comparing the intensities of the peaks of the β-Ti, α-Ti, TiB and TiB_2_ phases. Assuming Gaussian XRD peak shapes, the intensities of the peaks of different phases were calculated. The peak intensities of α-Ti, TiB and TiB_2_ were obtained to estimate the amount of TiB phase, and the detailed formulae are presented as follows:

(3) $$V_{TiB}=\frac{I_{TiB}}{I_{TiB}+\frac{R_{TiB}}{R_{\alpha Ti}}I_{\alpha Ti}+\frac{R_{TiB}}{R_{\beta Ti}}I_{\beta Ti}}$$

or

(4) $$V_{TiB}=\frac{I_{TiB}}{I_{TiB}+\frac{R_{TiB}}{R_{\alpha Ti}}I_{\alpha Ti}+\frac{R_{TiB}}{R_{TiB_{2}}}I_{TiB_{2}}}$$

In the above formulae, I is the peak intensity of a crystal face of a certain phase, and R is a parameter related to the angle and is normalised with respect to describing multiple crystallisation factors. By neglecting the temperature factor, it can be calculated as

(5) $$R=\frac{{\left|F_{hkl}\right|}^{2}pL}{V_{0}{}^{2}}$$

Here, V_0_ is the volume of the unit cell. F_hkl_ is the structure factor, p is the multiplicity factor and L is the Lorentz polarisation factor. If we know the peak intensities and parameter R of the α-Ti, TiB and TiB_2_ phases, the amounts of different phases in the Ti–TiB composite can be accurately calculated.

The calculated diffraction intensities were confirmed using Equation (6):

(6) $$I={\left|F_{hkl}\right|}^{2}pL$$

The multiplicity factors and Lorentz polarisation factors were determined using the standard data [16]. The structure factors were calculated based on the composition and coordinates of atoms in a crystal cell using Equation (7):

(7) $${\left|F\right|}^{2}=F\times F^{*}=\{\sum_{j=1}^{n}f_{j}e^{2\pi i(hx_{j}+ky_{j}+lz_{j})}\sum_{j=1}^{n}f_{j}e^{-2\pi i(hx_{j}+ky_{j}+lz_{j})}\}$$

where j = 1, 2, 3…n; f_j_ is an atomic scattering factor, and (x_j_, y_j_, z_j_) is the atomic position. The structure factors of TiB, α-Ti and TiB_2_ can be calculated using the standard data, while that of β-Ti cannot be calculated using the standard data because the exact atomic coordinate in the unit is unknown. In the present study, the β-Ti phase was generated by adding the β-Ti stabilising elements, Fe and Mo, and these Fe and Mo atoms replace the Ti atoms in the sublattice. Based on the assumption that the β-Ti stabilising atoms adopt a totally free arrangement, the average atomic scattering factor can be computed in terms of this regularity of mixtures. Thus, the average scattering factor (fav) can be written as follows:

(8) $$f_{av}=X_{Ti}f_{Ti}+X_{Fe}f_{Fe}+X_{Mo}f_{Mo}$$

where X_Ti_, X_Fe_ and X_Mo_ are the atomic fractions and f_Ti_, f_Fe_ and f_Mo_ are the corresponding atomic scattering factors of Ti, Fe and Mo atoms, respectively. The volume of the β-Ti unit cell was calculated using the lattice parameter (d = 0.32574 nm) calculated from the XRD spectrum of β-Ti. F_hkl_, p and L and the intensity data for the TiB, β-Ti, α-Ti and TiB_2_ phases based on the JCPDS files were listed in Table 1, Table 2, Table 3 and Table 4. These calculated diffraction intensities and the normalized integrated intensities measured from the experimental diffraction spectra and the crystal faces normalized according to the normalized dominant peak are also given in Table 1, Table 2, Table 3 and Table 4. The computed intensities are consistent with those in the JCPDS files. The intensities of the three dominant peaks of the computed and experimental data for β, α-Ti and TiB_2_ were also consistent with those in the JCPDS files. For TiB, the most dominant peak was that of the crystal face of (102)_TiB_, and the three dominant peaks in the computed data matched well with the experimental peaks. Thus, the amount of TiB based on F_hkl_ and R can be calculated using Equations (3) and (4). The non-overlapping peaks in this study were identified as (210)_TiB_, (102)_TiB_, (002)_α-Ti_, (101)_α-Ti_, (200)_β-Ti_, (001)_TiB2_ and (101)_TiB2_. Similarly, we calculated the amount of TiB_2_. The estimated volume fractions of TiB and TiB_2_ were averaged and shown in Table 5. The calculated TiB volume fractions were slightly higher than the targets in layers 2–4, and the deviations in layers 4 and 5 were less than 5% of the target volume fraction. In layers 5 and 6, the calculated TiB volume fractions were 76.5% and 73.0% and the TiB_2_ volume fractions were 2.2% and 16.0%, respectively. The target volume fraction of TiB was not achieved because of the presence of residual TiB_2_. When more TiB_2_ was retained in layer 6, the quantity of agglomerated TiB was reduced. Although TiB_2_ did not fully participate in the reaction with Ti in layers 5 and 6, the fractions of strengthening phases TiB and TiB_2_ were still more than those in layers 1–4. TiB_2_ is more stable than TiB at high temperatures or in the presence of excess boron in a Ti alloy matrix. The TiB_2_ phase was found in the sample fabricated to reach a target amount of 100% TiB by reacting Ti and B [15]. A similar effect was achieved by other methods involving self-propagating combustion synthesis at high temperatures [17,18].

### 3.2. Microstructure

**Figure 3:**
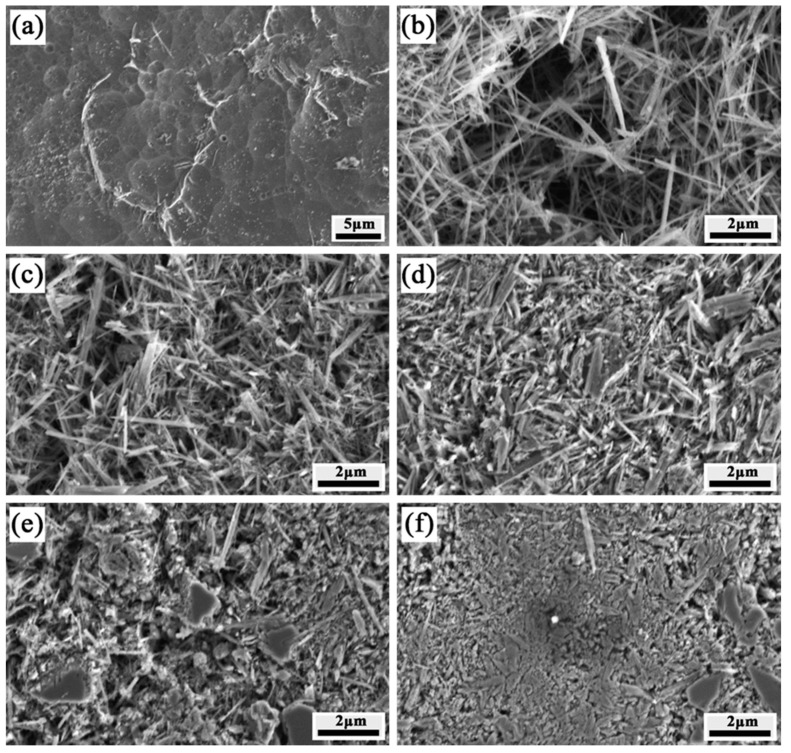
Micrographs of layers 1-6 of temperature gradient field sintering. (**a**) layer 1, (**b**) layer 2, (**c**) layer 3, (**d**) layer 4, (**e**) layer 5 and (**f**) layer 6.

In order to evaluate the accuracy of the calculation results of TiB volume fractions, the microstructures of FGM were observed for comparison with theoretical calculation result. Figure 3 exhibits the multifarious microstructures of the different layers in the FGM. The SEM micrograph of the Ti alloy matrix in Figure 3a shows the existence of equiaxial grains of β-Ti phase. In this design, the presence of FeMo powder forms a solid solution because Fe and Mo can diffuse into the Ti matrix to form a β-Ti phase. Occasionally, acicular TiB whiskers appeared in the grain boundaries. Figure 3b shows the morphology of layer 2, and randomly distributed acicular TiB whiskers presented in the titanium matrix. Figure 3c shows several agglomerated TiB whiskers in layer 3, which appear amid the acicular TiB whiskers in the layer for which the target amount of TiB is 40%. As shown in Figure 3d, there are two types of TiB features. Figure 3e,f show the microstructures in layers 5 and 6, wherein acicular, agglomerated TiB and unetched TiB_2_ coexist. The TiB_2_ content of the final layer is high compared to that of layer 5 and is consistent with the estimated TiB volume fraction. It can be seen that the content of TiB approximately accord with the result of calculations from observation of microstructure. This is not direct correlated with the theoretical calculations, but it indirectly reflects that the TiB distribution agrees with the result above.

### 3.3. Residual Stress Distributions in the FGM

The residual stress in the FGM was calculated based on the elastic axial symmetry model using the finite element simulation. The Ti side was assumed to be at 900 °C and the TiB side at 1500 °C during sintering, and the residual thermal stress was generated under elastic conditions during cooling. The Halpin–Tsai Equation was used to calculate the elastic moduli of each layer for discontinuously reinforced composites as follows [19]:

(9) $$E_{c}=\frac{E_{m}(1+2sqV_{p})}{1-qV_{p}}$$

The following Equation was used to obtain parameter q:

(10) $$q=\frac{E_{p}/E_{m}-1}{E_{p}/E_{m}+2s}$$

*E_m_* is the Young’s modulus of the Ti alloy matrix, *Ep* is the reinforcement and *V_p_* and *s* are the volume and aspect ratio of the reinforcement, respectively. The Young’s modulus of β-Ti was considered as 115 GPa based on the research of Feng et al. [20]. Herein, the elastic moduli were calculated by considering the moduli of TiB and TiB_2_ as 550 GPa [21,22]. Based on previous work, the aspect ratios of the TiB whiskers in the layers were 20–7.5 and that in the TiB-rich layer was assumed as 7.5 [15]; this adequately explains the variation in elastic modulus versus the amount of TiB, although the microstructure contained a bimodal TiB distribution [23]. Turner’s equation was used to calculate the CTEs of the different layers in composites with reinforcing particles,

(11) $$\alpha_{c}=\frac{\alpha_{m}V_{m}E_{m}+\alpha_{p}V_{p}E_{p}}{V_{m}E_{m}+V_{p}E_{p}}$$

where *α_m_* and *α_p_* are the CTE values, *V_m_* and *V_p_* are the volumes and *E_m_* and *E_p_* are the moduli. The subscripts *m* and *p* indicate the matrix and reinforcement, respectively. To calculate the residual stresses in the FGM, the CTE values for Ti, TiB and TiB_2_ at room temperature were considered as 9.0 × 10^−6^/K, 7.15 × 10^−6^/K and 6.2 × 10^−6^/K, respectively. The calculated CTEs for the layers in the FGM are given in Table 5. 

**Figure 4:**
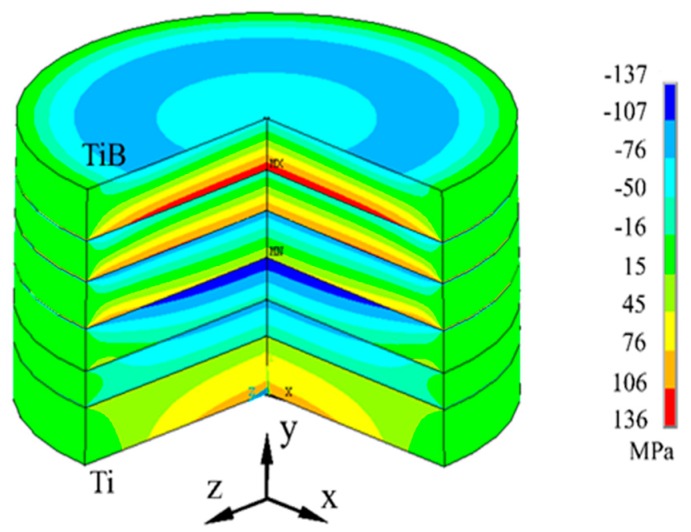
3-D Geometrical model and residual stress distribution of the Ti–TiB–TiB_2_ FGM.

**Figure 5:**
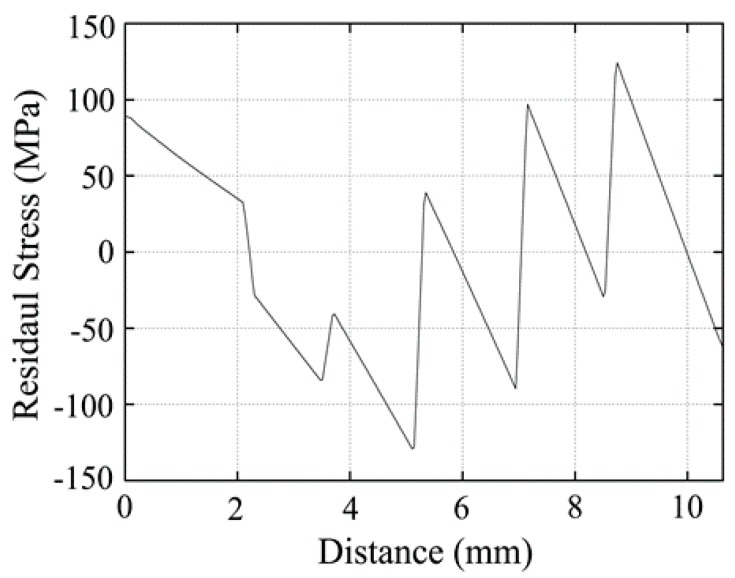
Calculated axial stress in the direction of the radial centre of the FGM sintered by TGF.

**Figure 6:**
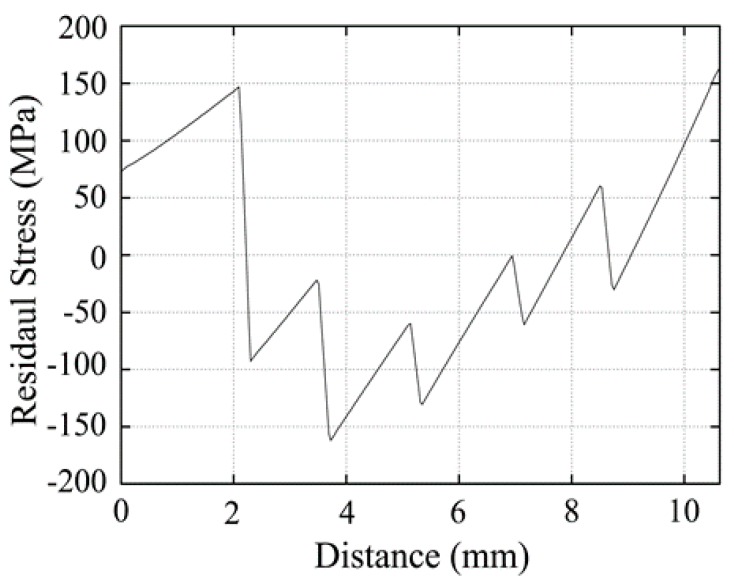
Calculated axial stress in the direction of the radial centre of the FGM uniformly sintered at 1100 °C.

Figure 4 shows the calculated residual stress in the TGF sintered FGM. Both the maximum residual stresses are distributed along the axial direction of the cylinder sample, and the sample bends to the TiB side. Since the TiB-rich side cooled from 1500 °C, which was hotter than the Ti-rich side, the contraction of the TiB-rich layer was greater than that of the Ti-rich layer, causing the TGF sintered sample to bend to the TiB side. Figure 5 shows the radial (X-axis) stress distributions along the sample (Y-axis) in the TGF sintered FGM; this indicates that the residual stresses changed from tensile to compressive as the distance from the Ti-rich side increased in the three TiB-rich layers. The maximum tensile stress is 124 MPa and the maximum compressive stress is 129 MPa. Meanwhile, the residual stress generated in the FGM with the same layer structure, sintered at a uniform temperature of 1100 °C, was calculated. Figure 6 shows the radial (X-axis) stress distributions along the sample (Y-axis) in the uniformly sintered FGM. The TiB and Ti-rich layers are under tension, while other layers are generally under compression; thus, the stress distribution is different from that of the TGF sintered FGM. The maximum tensile stress is 164 MPa and the maximum compressive stress is 162 MPa. It is noteworthy that even though the temperature of the TiB-rich side was approximately 1500 °C during the TGF sintering, both the residual tensile and compressive stresses were less than those of uniformly sintered FGM at 1100 °C. Thus, the Ti-rich side was sintered at low temperature and the TiB-rich side was sintered at high temperature in the FGM, and relatively low thermal residual stress can be achieved using the TGF sintering method. This method is considered suitable for sintering many analogous metal and ceramic matrix FGMs.

## 4. Conclusions

Ti matrix composite with a polylaminate structure was successfully fabricated via SPS. A TGF was obtained during the FGM sintering. The calculated TiB volume fractions were slightly higher than the target volume fractions in layers 2–4 and lower than the target volume fractions in layers 5 and 6. In layers 5 and 6, the calculated TiB volume fractions were 76.5% and 73.0%, and the TiB_2_ volume fractions were 2.2% and 16.0%, respectively. An equiaxial β-Ti phase in layer 1, TiB and Ti phases in layers 2–4 and a TiB_2_ phase in layers 5–6 were observed. Acicular and agglomerated TiB were present in the FGM. The residual stress distributions in the FGM were calculated based on an elastic axial symmetry model using the finite element method. Both the maximum residual stresses were distributed along the axial direction of the cylinder sample. The residual stresses of the radial (X-axis) and along the sample (Y-axis) in the TGF sintered FGM changed from tensile to compressive as the distance from the Ti-rich side increased in the three TiB-rich layers. The maximum tensile stress was 124 MPa and the maximum compressive stress was 129 MPa. The maximum tensile and compressive residual stresses, generated in the FGM with the same layer sintered at a uniform temperature of 1100 °C, were 164 and 162 MPa, respectively. A relatively low thermal residual stress was achieved by the TGF sintering method. This method is considered suitable for sintering many analogous metals and ceramic matrix FGMs.

## Figures and Tables

**Table 1:** Normalized integrated X-ray line intensities for the TiB peaks.

hkl	2θ(Deg)	d(A)	|F_hkl_|^2^	L	p	I_TiB_ in JCPDS	I_TiB_ Computed	I_TiB_ in XRD	R Calculated
101	24.502	3.633	906	41.20	4	20	82	14	20.46
200	29.252	3.053	2501	29.08	2	32	80	17	19.92
201	35.293	2.543	1089	19.14	4	80	46	11	11.42
111	38.368	2.346	718	15.80	8	80	50	8	12.44
210	41.799	2.161	1142	13.09	4	64	33	84	8.19
102	42.229	2.140	3549	12.81	4	100	100	100	24.91
211	46.422	1.956	713	10.35	8	40	32	-	8.09
301	48.887	1.863	377	9.23	4	56	8	-	1.91
112	52.113	1.755	722	7.92	8	40	25	30	6.27
020	60.598	1.528	4315	5.65	2	32	2	-	6.68
401	63.694	1.461	452	5.08	4	28	5	-	1.26
312	68.944	1.362	2508	4.27	8	72	47	54	11.73

**Table 2:** Normalized integrated X-ray line intensities for the β-Ti peaks.

hkl	2θ(Deg)	d(A)	|F_hkl_|^2^	L	p	I_β-Ti_ in JCPDS	I_β-Ti_ Computed	I_β-Ti_ in XRD	R Calculated
110	38.482	2.3375	1277	15.80	12	100	100	100	202.68
200	55.543	1.6532	907	6.86	6	12	15	9	31.25
211	69.607	1.3496	646	4.19	24	17	27	27	54.38
220	82.447	1.1689	639	3.12	12	4	10	7	20.03
310	94.927	1.0454	462	2.74	24	5	13	6	25.43
222	107.628	0.9544	462	2.84	8	1	4	-	8.79

**Table 3:** Normalized integrated X-ray line intensities for the α-Ti peaks.

hkl	2θ(Deg)	d(A)	|F_hkl_|^2^	L	p	I_α-Ti_ in JCPDS	I_α-Ti_ Computed	I_α-Ti_ in XRD	R Calculated
100	35.063	2.557	34161	19.38	6	30	23	17	27.38
002	38.402	2.342	37134	15.80	2	26	25	28	29.77
101	40.150	2.244	151026	14.28	12	100	100	100	121.06
102	53.008	1.726	19115	7.65	12	19	13	-	15.32
110	62.960	1.475	25887	5.18	6	17	17	18	20.75
103	70.657	1.332	214175	4.07	12	16	14	-	17.17
200	74.263	1.276	0	3.70	6	2	0	-	0
112	76.294	1.247	24626	3.51	12	16	16	-	19.74

**Table 4:** Normalised intensities of X-ray diffraction peaks for TiB_2_.

hkl	2θ(Deg)	d(A)	|F_hkl_|^2^	L	p	I_TiB2_ in JCPDS	I_TiB2_ Computed	I_TiB2_ in XRD	R Calculated
001	27.598	3.2295	110	32.31	2	22	19	19	10.78
100	34.133	2.6246	208	20.38	6	55	67	44	38.60
101	44.438	2.0370	279	11.43	12	100	100	100	58.05
002	56.992	1.6145	339	6.48	2	12	11	10	6.67
110	61.106	1.5153	402	5.52	6	27	35	37	20.207
102	68.134	1.3751	86	4.37	12	16	12	-	6.847
111	68.328	1.3717	42	4.35	-	18	-	-	-
200	71.895	1.3121	108	3.94	6	7	7	-	3.88
201	78.642	1.2156	190	3.35	12	16	20	9	11.58

**Table 5:** Target and calculated TiB volume fractions and coefficient of thermal expansion (CTE) values for different layers in the functionally graded material (FGM).

**Layers**	**Target Volume Fraction of TiB(%)**	**Calculated Volume Fraction (%)**		**Estimated Composition (wt%)**			**CTE(/K)**
		**TiB**	**TiB_2_**	**TiB**	**TiB_2_**	**Ti**	
Layer1	0	0	0	0	0	100	9.00
Layer2	20	24.4	0	23.3	0	76.7	7.89
Layer3	40	45.7	0	44.2	0	55.8	7.52
Layer4	60	61.6	0	60.2	0	39.8	7.36
Layer5	80	76.5	2.2	75.5	2.2	22.3	7.22
Layer6	100	73.0	16.0	72.5	15.9	11.6	7.03
